# Complex Rayleigh Waves in Nonhomogeneous Magneto-Electro-Elastic Half-Spaces

**DOI:** 10.3390/ma14041011

**Published:** 2021-02-21

**Authors:** Ke Li, Shuangxi Jing, Jiangong Yu, Bo Zhang

**Affiliations:** School of Mechanical and Power Engineering, Henan Polytechnic University, Jiaozuo 454003, China; like@hpu.edu.cn (K.L.); jsx@hpu.edu.cn (S.J.); bozhanghpu@163.com (B.Z.)

**Keywords:** complex rayleigh waves, laguerre orthogonal polynomial, nonhomogeneous, magneto-electro-elastic, half-space

## Abstract

The complex Rayleigh waves play an important role in the energy conversion efficiency of magneto-electro-elastic devices, so it is necessary to explore the wave propagation characteristics for the better applications in engineering. This paper modifies the Laguerre orthogonal polynomial to investigate the complex Rayleigh waves propagating in nonhomogeneous magneto-electro-elastic half-spaces. The improved method simplifies the calculation process by incorporating boundary conditions into the constitutive relations, shortens the solving time by transforming the solution of wave equation to an eigenvalue problem, and obtains all wave modes, including real and imaginary and complex wavenumbers. The three-dimensional curves of full frequency spectrum and phase velocities are presented for the better description of the conversion from complex Rayleigh wave modes to real wave ones; besides, the displacement distributions, electric and magnetic potential curves are obtained in thickness and propagation directions, respectively. Numerical results are analyzed and discussed elaborately in three cases: variation of nonhomogeneous coefficients, absence of magnetism, and absence of electricity. The results can be used to optimize and fabricate the acoustic surface wave devices of the nonhomogeneous magneto-electro-elastic materials.

## 1. Introduction

The past few decades have seen considerable time and effort invested in studying the mechanics of magneto-electro-elastic composites. With their particular properties, such as the mutual conversion of magneto-electro-elastic energy [1], these materials are widely applied in sensors, ultrasonic nondestructive evaluation, and vibration control devices [2,3,4,5]. They are so promising for many applications that they have become the next generation multi-functional materials [6]. To improve their thermal and corrosive resistance, the research has gradually expanded to the field of nonhomogeneous material; thereafter, the study of the waves propagating in nonhomogeneous magneto-electro-elastic composites has received more and more attention [7,8], and some researchers have successfully fabricated an actuator with small mechanical damping and heat generation by using functionally graded piezoelectric material [9].

The study on the propagation behaviors in these materials is so important and urgent that many researchers have been working in this field. Pang et al. [10] investigated Rayleigh-type surface waves in a piezoelectric-piezomagnetic layered half-space. Wei et al. [11] explored the propagation characteristics of shear horizontal surface waves propagating in a layered piezoelectric-piezomagnetic half-space and discussed the sources that effect on the dispersion curves and phase velocities. For investigating the Love waves propagation in layered magneto-electro-elastic half-space, Bou Matar et al. [12] combined the Legendre and Laguerre polynomial technique to obtain their solutions. Du [13] not only investigated the Love waves propagating in layered magneto-electro-elastic half-space with initial stress and viscous liquid, respectively [14,15], but also studied the propagation of the Rayleigh waves in a piezoelectric-piezomagnetic layered half-space affected by a biasing electric field [16] and a biasing magnetic field [17], respectively. Their results show that mechanical displacement and phase velocity are greatly effected by the biasing electric and magnetic fields. In 2016, Ezzin et al. [18] discussed the influence of material properties on phase velocity and analyzed the dispersion characteristic, through examining the Love waves propagating in a piezoelectric material layer on a piezomagnetic half-space.

The efforts mentioned above are limited in the magneto-electro-elastic half-space without involving the nonhomogeneous materials, in fact, the unprecedented progress has been achieved in nonhomogeneous magneto-electro-elastic half-space. Zhang et al. [19], using the Wentzel-Kramers-Brillouin approximate method, investigated the Love wave propagation in layered inhomogeneous magneto-electro-elastic half-spaces with initial stress. Singh and Bokne [20] analyzed the propagation of shear horizontal waves in a functionally graded magnetoelectric half-space and obtained the dispersion relations. Ezzin et al., using the ordinary differential equation and stiffness matrix methods, studied the propagation of the Rayleigh [21] and Love [22] waves on a piezomagnetic half-space covered with a functionally graded piezoelectric half-space. In terms of pure half-space (without overlay) with electro-magneto-elastic materials, Feng et al. [23] studied the conditions that effect on phase velocities of Rayleigh wave. Yang et al. investigated the Rayleigh waves propagating in a magneto-electric half-space rotating at a constant angular rate about a fixed axis. In 2014, Zhang [24] studied the Rayleigh wave’s characteristics in a magneto-electro-elastic half-space with initial stress by using the quasistatic approximation and linearity assumption. Through assuming the material properties are space dependent, the Love wave propagation in non-homogeneous electro-magneto-elastic half-space was explored by employing the mathematical approach [25]. Later on, it was found that in different cross sections the surface wave velocity can have significant change due to the rotary symmetry of crystal [26].

Recently, the study of complex wave propagation has received considerable attention because it is found that some complex wave modes can be converted or degenerated to propagating waves [27,28], and they are very suitable to fabricate the high-performance surface acoustic wave devices operating in the GHz range of frequencies [29]. Moreover, the complex waves are full of promise in the field of non-destructive testing [30,31] and can be used to develop high sensitive sensors [32]. For propagating waves, obtaining their solution remains simple because the wavenumbers are real. But, when the wave equations are solved in complex space, it will give the solution containing purely real, purely imaginary and complex values with respect to wavenumbers. It is well known that the purely real solutions represent propagating waves. For the purely imaginary and complex solutions, they represent evanescent wave modes. The former are exponentially damped with propagating distance, and the latter’s decay follows a damped sinusoidal distribution. Some theoretical and experimental researches in terms of the propagation of complex waves have been carried out [33]. A recent study on the complex waves was conducted by Giurgiutiu et al. [34], who investigated the propagating, evanescent, and complex wavenumber guided waves in high-performance composites. Although some articles have explored the complex waves [35,36], they are very rarely used to discuss the complex waves in nonhomogeneous magneto-electro-elastic half-space, especially in the terms of complex Rayleigh waves.

In this paper, an improved polynomial expansion approach is given for the solutions of the complex Rayleigh waves propagating in nonhomogeneous magneto-electro-elastic half-spaces. The Laguerre polynomial has many advantages, e.g., ① its orthogonality can be used to reduce the time-consuming in calculation; ② it is simple to program; and ③ its solution is noniterative. So, it has been employed to obtain the solution of surface waves propagating in half-spaces for decades [37]. However, the conventional method can only get the real solutions. This paper will transform the conventional Laguerre orthogonal polynomial to the eigenvalue problem of wavenumbers and define the boundary conditions using the Heaviside step function; by solving the characteristic matrix, the eigenvalues give the solution of propagating, evanescent, and complex wavenumber waves in nonhomogeneous magneto-electro-elastic half-spaces; moreover, the eigenvectors are obtained to present the mechanical displacement, electric, and magnetic potentials. For examining the effect of different material constants on the complex Rayleigh waves, three cases are taken account of: nonhomogeneous magneto-electro-elastic half-space, nonhomogeneous piezoelectric half-space, and nonhomogeneous piezomagnetic half-space, and their results are compared and discussed in detail. This paper also investigated the effect of the nonhomogeneous variation on the complex Rayleigh wave propagation characteristics. Furthermore, the 3D dispersion curves are obtained for discussing the cut-off frequencies, and the phase velocity curves are given for exploring the wave conversion.

## 2. Theoretical Formulation and Solution

### 2.1. Basic Equations

**Figure 1:**
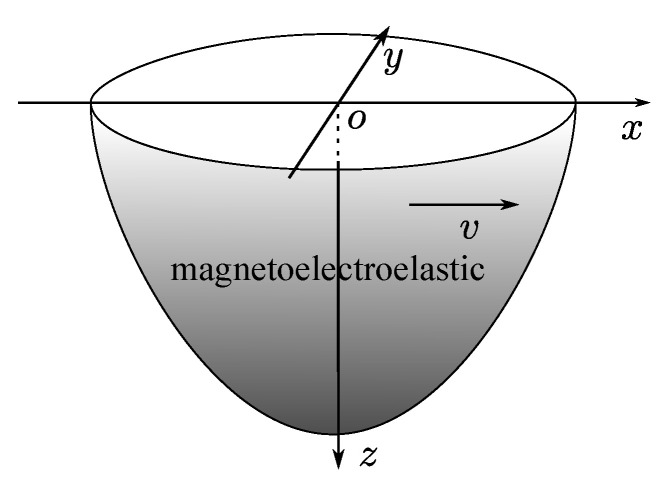
A nonhomogeneous magneto-electro-elastic half-space showing in the coordinate system.

Consider an orthotropic nonhomogeneous magneto-electro-elastic half-space, where the material coefficients vary continuously along the *z* direction. The Cartesian coordinate system (as shown in Figure 1) is designated as follows: the upper surface of the half-space is set as the horizontal coordinate plane x−y; the z axis is downward and perpendicular to the x−y plane; the half-space occupies the region $\left[0,\infty \right)$ in the *z* direction. Rayleigh surface waves propagate in the *x*-axis direction.

Let $u_{i}$ represent the components of particle displacement in the *i*th direction, and ρ be the mass density. When the electric and magnetic sources are absent, the governing equations in the half-space are

(1) $$\begin{array}{cc} \sigma_{ij,j} & =\rho {\ddot{u}}_{i}\\ D_{i,i} & =0\;(i,j=x,y,z)\\ B_{i,i} & =0 \end{array},$$

in which ${}_{.j}$ denotes differentiation with $x_{j}$, the ${\ddot{u}}_{i}$ represents the second derivative of $u_{i}$ over time, *B* is the magnetic induction, *D* is the electric displacement, and σ denotes the stress. The Einstein summation notation is applied here. For a linear, anisotropic and coupled mechanical-electric-magnetic material medium, the constitutive equations [38,39] are

(2) $$\begin{array}{cccc} \sigma_{ij} & =c_{ijkl}\varepsilon_{kl} & -e_{kij}E_{k} & -q_{kij}H_{k}\\ D_{i} & =e_{ikl}\varepsilon_{kl} & +\epsilon_{ik}E_{k} & +d_{ik}H_{k}\;\left(i,j,k,l=x,y,z\right)\\ B_{i} & =q_{ikl}\varepsilon_{kl} & +d_{ik}E_{k} & +\mu_{ik}H_{k} \end{array},$$

where $E_{k}$, $H_{k}$, and $\varepsilon_{kl}$ are the electric field, magnetic field, and strain, respectively; $\epsilon_{ik}$, $d_{ik}$, and $\mu_{ik}$ are, respectively, the dielectric, magnetoelectric, and permeability coefficients; and $e_{kij}$, $q_{kij}$, and $c_{ijkl}$ are the piezoelectric, piezomagnetic, and elastic coefficients, respectively.

Based on the elasticity theory and the quasi-static Maxwell equation, the strain, electric, and magnetic field are related to the mechanical displacement $u_{i}$, the electric potential ϕ, and magnetic potential φ, as follows:

(3) $$\begin{array}{cc} \varepsilon_{ij} & =\frac{1}{2}\left(u_{i,j}+u_{j,i}\right)\\ E_{i} & =-\phi_{,i}\;\;\left(i,j=x,y,z\right)\\ H_{i} & =-\varphi_{,i} \end{array}.$$

We just consider the waves propagating in the nonhomogeneous magneto-electro-elastic half-space polarized along the *z*-axis direction. The upper surface are magnetically short and electrically open [24] in the present paper. Therefore, the mechanical and electric-magnetic boundary conditions at the upper surface are

(4) $$\sigma_{zz}=0,\;\sigma_{xz}=0,\;D_{z}=0,\;B_{z}=0\;\left(at\;z=0\right).$$

So, combining Equations (1) and (4) will obtained the solution of the Rayleigh waves propagating in the magneto-electro-elastic nonhomogeneous half-space.

### 2.2. Solution of the Problem

In this study, the variables of field are independent of *y* because the Rayleigh waves propagate along *x*-axis; therefore, Equation (1) can be written:

(5) $$\begin{array}{cccc} \frac{\partial \sigma_{xx}}{\partial x} & +\frac{\partial \sigma_{xz}}{\partial z} & = & \rho \frac{\partial^{2}u_{x}}{\partial t^{2}}\\ \frac{\partial \sigma_{xz}}{\partial x} & +\frac{\partial \sigma_{zz}}{\partial z} & = & \rho \frac{\partial^{2}u_{z}}{\partial t^{2}}\\ \frac{\partial D_{x}}{\partial x} & +\frac{\partial D_{z}}{\partial z} & = & 0\\ \frac{\partial B_{x}}{\partial x} & +\frac{\partial B_{z}}{\partial z} & = & 0 \end{array}.$$

By introducing the unit step function h(z):

(6) $$h\left(z\right)=\left\{\begin{array}{cc} 1, & z\geq 0;\\ 0, & elsewhere \end{array}\right..$$

The above boundary conditions described in Equation (4) can be automatically incorporated into Equation (2), which leads to

(7) $$\begin{array}{cc} \sigma_{xx} & =c_{11}\varepsilon_{xx}+c_{13}\varepsilon_{zz}-e_{31}E_{z}-q_{31}H_{z}\\ \sigma_{zz} & =\left(c_{13}\varepsilon_{xx}+c_{33}\varepsilon_{zz}-e_{33}E_{z}-q_{33}H_{z}\right)h\left(z\right)\\ \sigma_{xz} & =\left(c_{44}\varepsilon_{zx}+c_{44}\varepsilon_{xz}-e_{15}E_{x}-q_{15}H_{x}\right)h\left(z\right)\\ D_{x} & =e_{15}\varepsilon_{zx}+e_{15}\varepsilon_{xz}+\epsilon_{11}E_{x}+d_{11}H_{x}\\ D_{z} & =\left(e_{31}\varepsilon_{xx}+e_{33}\varepsilon_{zz}+\epsilon_{33}E_{z}+d_{33}H_{z}\right)h\left(z\right)\\ B_{x} & =q_{15}\varepsilon_{zx}+q_{15}\varepsilon_{xz}+d_{11}E_{x}+\mu_{11}H_{x}\\ B_{z} & =\left(q_{31}\varepsilon_{xx}+q_{33}\varepsilon_{zz}+d_{33}E_{z}+\mu_{33}H_{z}\right)h\left(z\right) \end{array}.$$

Assuming that the material properties are functions of depth, then the mechanical and electric-magnetic coefficients become the functions related to f(z) (it is assumed that they all are controlled by a same function for the convenience of expression). In practice, every material coefficient can be applied distinct continuous function, such as $c\left(z\right)=c\;\cdot \;f_{1}\left(z\right),e\left(z\right)=e\;\cdot \;f_{2}\left(z\right)\cdots$), and they can be defined as

(8) $$\begin{array}{ccc} c(z)=c\;\cdot \;f(z) & e(z)=e\;\cdot \;f(z) & q(z)=q\;\cdot \;f(z)\\ \epsilon (z)=\epsilon \;\cdot \;f(z) & d(z)=d\;\cdot \;f(z) & \mu (z)=\mu \;\cdot \;f(z)\\ \rho (z)=\rho \;\cdot \;f(z) & \; \end{array},$$

where e,q,ϵ,d,μ, and *c* are, respectively, the piezoelectric, piezomagnetic, dielectric, magnetoelectric permeability, and elastic constants in the homogenous material.

Assuming a Rayleigh surface wave propagating along *x*-axis, then the fields of mechanical displacement, electric, and magnetic potential can be written in the following form:

(9a) $$\begin{array}{cccc} & u_{x}\left(x,z,t\right) & & =e^{i(kx-\omega t)}U\left(z\right) \end{array}$$

(9b) $$\begin{array}{cccc} & u_{z}\left(x,z,t\right) & & =e^{i(kx-\omega t)}W\left(z\right) \end{array}$$

(9c) $$\begin{array}{cccc} & \phi (x,z,t) & & =e^{i(kx-\omega t)}X\left(z\right) \end{array}$$

(9d) $$\begin{array}{cccc} & \varphi (x,z,t) & & =e^{i(kx-\omega t)}Y\left(z\right). \end{array}$$

Firstly, substituting Equations (7)–(9a–d) into Equation (5), and then reorganizing all these equations according to the orders of wavenumbers and categorizing them by the terms of $k^{2}$, $k^{1}$, and $k^{0}$, therefore, Equation (5) can be expressed as:

(10) $$\begin{array}{c} -c_{11}f\left(z\right)Uk^{2}{|}_{k^{2}}^{\mathrm{term}}\\ +\left[{\left(\left(c_{44}W+e_{15}X+q_{15}Y\right)h\left(z\right)f\left(z\right)\right)}^{\prime }+{\left(c_{13}W+e_{31}X+q_{31}Y\right)}^{\prime }f\left(z\right)\right]{ik|}_{k^{1}}^{\mathrm{term}}\\ +c_{44}{\left(U^{\prime }f\left(z\right)h\left(z\right)\right)}^{\prime }{|}_{k^{0}}^{\mathrm{term}}=-\rho \omega^{2}Uf\left(z\right),\\ -\left(c_{44}W+e_{15}X+q_{15}Y\right)f\left(z\right)h\left(z\right)k^{2}{|}_{k^{2}}^{\mathrm{term}}\\ +\left[{\left(c_{13}Uh\left(z\right)\right)}^{\prime }+c_{44}U^{\prime }f\left(z\right)h\left(z\right)\right]{ik|}_{k^{1}}^{\mathrm{term}}\\ +{\left[{\left(c_{33}W+e_{33}X+q_{33}Y\right)}^{\prime }f\left(z\right)h\left(z\right)\right]}^{\prime }{|}_{k^{0}}^{\mathrm{term}}=-\rho \omega^{2}Wf\left(z\right),\\ \left(-e_{15}W+\epsilon_{11}+d_{11}Y\right)f\left(z\right)k^{2}{|}_{k^{2}}^{\mathrm{term}}\\ +\left[{\left(e_{31}Uh\left(z\right)f\left(z\right)\right)}^{\prime }+e_{15}U^{\prime }f\left(z\right)\right]{ik|}_{k^{1}}^{\mathrm{term}}\\ +{\left[{\left(e_{33}W-\epsilon_{33}X-d_{33}Y\right)}^{\prime }f\left(z\right)h\left(z\right)\right]}^{\prime }{|}_{k^{0}}^{\mathrm{term}}=0,\\ \left(-q_{15}W+d_{11}X+\mu_{11}Y\right)f\left(z\right)k^{2}{|}_{k^{2}}^{\mathrm{term}}\\ +\left[{\left(q_{31}Uh\left(z\right)f\left(z\right)\right)}^{\prime }+q_{15}U^{\prime }f\left(z\right)\right]{ik|}_{k^{1}}^{\mathrm{term}}\\ +{\left[{\left(q_{33}W-d_{33}X-\mu_{33}Y\right)}^{\prime }f\left(z\right)h\left(z\right)\right]}^{\prime }{|}_{k^{0}}^{\mathrm{term}}=0 \end{array}$$

where the prime mark (${}^{\prime }$) represents a derivative, and using it twice denotes the second derivative. When the Laguerre orthogonal polynomial series are introduced, U,W,X, and *Y* can be written as:

(11) $$\begin{array}{cc} U\left(z\right)=\sum_{m=0}^{\infty }p_{m}^{1}Q_{m}\left(z\right) & \;W\left(z\right)=\sum_{m=0}^{\infty }p_{m}^{2}Q_{m}\left(z\right),\\ X\left(z\right)=\sum_{m=0}^{\infty }r_{m}^{1}Q_{m}\left(z\right) & \;Y\left(z\right)=\sum_{m=0}^{\infty }r_{m}^{2}Q_{m}\left(z\right), \end{array}$$

in which, $p_{m}^{i}$ and $r_{m}^{i}$ (i=1,2) represent expansion coefficients and

(12) $$Q_{m}\left(z\right)=Exp\left(-\frac{z}{2}\right)\frac{L_{m}\left(z\right)}{m!},$$

where $L_{m}\left(z\right)$ is the *m*th order Laguerre polynomial, and functions $Q_{m}\left(z\right)$ are orthonormal and complete from 0 to *∞*, so appropriate for calculating in semi-infinite interval. Even though *m* can be any non-negative integer (0,1,2,3,⋯), practically, the summation of Equation (11) has halted at some value m=M because the solutions have converged well within a finite number of terms.

To begin with, substituting Equation (11) into Equation (10), and then using the complex conjugate $Q_{j}^{*}\left(z\right)$ to multiply the obtained equations (*j* are integers from 0 to infinity), will lead to 4×(M+1) equations because any equation in Equation (10) will give (M+1) equations. Thirdly, integrate the product over *z* in the range $\left[0,\infty \right)$, and rearrange them according to $k^{2},k^{1}$, and $k^{0}$. Last, their matrix representation is achieved, as follows:

(13) $$k^{2}\mathrm{AP}+k\mathrm{BP}+\mathrm{CP}=\omega^{2}\mathrm{MP},$$

in which the vector and the square matrices can be expressed as the following Equation (14), and their elements can be obtained from Equation (10).

(14) $$\begin{array}{cccc} A & =\left[\begin{array}{cccc} A_{11}^{j,m} & A_{12}^{j,m} & A_{13}^{j,m} & A_{14}^{j,m}\\ A_{21}^{j,m} & A_{22}^{j,m} & A_{23}^{j,m} & A_{24}^{j,m}\\ A_{31}^{j,m} & A_{32}^{j,m} & A_{33}^{j,m} & A_{34}^{j,m}\\ A_{41}^{j,m} & A_{42}^{j,m} & A_{43}^{j,m} & A_{44}^{j,m} \end{array}\right]\; & B & =\left[\begin{array}{cccc} B_{11}^{j,m} & B_{12}^{j,m} & B_{13}^{j,m} & B_{14}^{j,m}\\ B_{21}^{j,m} & B_{22}^{j,m} & B_{23}^{j,m} & B_{24}^{j,m}\\ B_{31}^{j,m} & B_{32}^{j,m} & B_{33}^{j,m} & B_{34}^{j,m}\\ B_{41}^{j,m} & B_{42}^{j,m} & B_{43}^{j,m} & B_{44}^{j,m} \end{array}\right]\\ C & =\left[\begin{array}{cccc} C_{11}^{j,m} & C_{12}^{j,m} & C_{13}^{j,m} & C_{14}^{j,m}\\ C_{21}^{j,m} & C_{22}^{j,m} & C_{23}^{j,m} & C_{24}^{j,m}\\ C_{31}^{j,m} & C_{32}^{j,m} & C_{33}^{j,m} & C_{34}^{j,m}\\ C_{41}^{j,m} & C_{42}^{j,m} & C_{43}^{j,m} & C_{44}^{j,m} \end{array}\right]\; & M & =\left[\begin{array}{cccc} M_{11}^{j,m} & 0 & 0 & 0\\ 0 & M_{22}^{j,m} & 0 & 0\\ 0 & 0 & 0 & 0\\ 0 & 0 & 0 & 0 \end{array}\right] \end{array}$$

$$P={\left[p_{0}^{1},p_{1}^{1},\cdots ,p_{M}^{1},p_{0}^{2},p_{1}^{2},\cdots ,p_{M}^{2},r_{0}^{1},r_{1}^{1},\cdots ,r_{M}^{1},r_{0}^{2},r_{1}^{2},\cdots ,r_{M}^{2}\right]}^{T}.$$

To find the solution of wave number *k* in Equation (13), here, we introduce a new column vector N:

(15) $$N=\left[\begin{array}{c} n_{1}\\ n_{2}\\ n_{3}\\ n_{4} \end{array}\right]=k\left[\begin{array}{c} p^{1}\\ p^{2}\\ r^{1}\\ r^{2} \end{array}\right]=kP.$$

After inserting Equation (15) into Equation (13), the resulting equations can be rewritten as:

(16) $$k\mathrm{AN}+\mathrm{BN}+\mathrm{CP}=\omega^{2}\mathrm{MP}.$$

Separating *k* and P in Equation (16), the result becomes:

(17) $$A^{-1}\left(\omega^{2}M-C\right)P-A^{-1}\mathrm{BN}=kN.$$

Considering N=kP in Equation (15) and introducing an identity matrix I, thus, Equation (17) becomes:

(18) $$\left[\begin{array}{cc} 0 & I\\ A^{-1}\left(\omega^{2}M-C\right) & -A^{-1}B \end{array}\right]\left[\begin{array}{c} P\\ N \end{array}\right]=k\left[\begin{array}{c} P\\ N \end{array}\right],$$

where I has the same dimension as A. Letting

$$R=\left[\begin{array}{c} P\\ N \end{array}\right],$$

finally, Equation (18) can be further simplified to:

(19) $$\left[\begin{array}{cc} 0 & I\\ A^{-1}\left(\omega^{2}M-C\right) & -A^{-1}B \end{array}\right]R=kR.$$

Equation (19) indicates that the wave number *k* is an eigenvalue, and R is an eigenvector corresponding to the *k* in this equation. So, giving a series of angular frequency values ω, a series of corresponding wave numbers *k* will be generated in the form of eigenvalues, which also can give the phase velocity with ease. The field distributions can be calculated by the eigenvectors R. As can be seen, this equation considers the angular frequency ω as independent variables, and *k* as the eigenvalues. The wavenumber of the equation exists in three forms, namely $k^{2}$, $k^{1}$, and $k^{0}$. As a result, the equation can present all kinds of solutions, including the propagating and non-propagating wave numbers.

## 3. Numerical Results and Discussion

In this section, the propagation behaviors of complex Rayleigh waves in the nonhomogeneous magneto-electro-elastic half-space are studied numerically based on the formulations derived from the previous section. The material properties (at z=0) can be seen in Table 1, which vary gradually in the thickness direction, and the nonhomogeneous function $f\left(z\right)={\left(1+z\right)}^{n}$ in Equation (8), where *n* is the nonhomogeneous coefficient. The mass density ρ is $7500\;kg/m^{3}$ at z=0. The material constants in Table 1 are used for all examples, except for the section of approach validation, which used the material lead zirconate titanate (PZT-4).

### 3.1. Approach Validation

**Figure 2:**
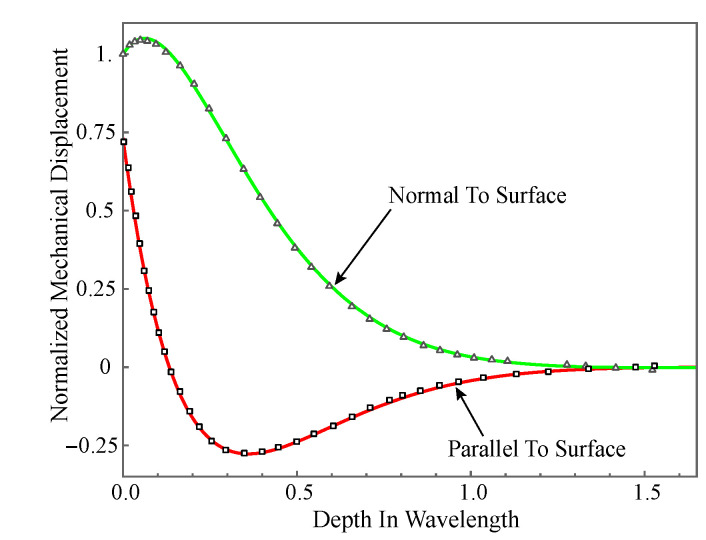
Normalized mechanical displacement amplitude distribution: solid curves are our results, and dotted lines are from literature.

The verification for the present approach is carried out in two parts: comparing the mechanical displacement with earlier works and validating the boundary conditions. To the authors’ knowledge, so far, the complex waves in nonhomogeneous magneto-electro-elastic half space have not been studied, so, here, we only present the displacement amplitude distribution in homogeneous PZT-4 half-space to make comparison with the available numerical results. The used material properties are taken from the literature [41]. The mechanical displacement amplitude distributions for the purely real solutions are given in Figure 2 for the order truncation M=10. The curves in this figure represent normalized mechanical displacement ($u/w_{0}$ and $w/w_{0}$). The solid curves are obtained from the present paper when the wavenumber k=2, and the dotted lines are taken from the literature [42]. Obviously, the excellent agreement between them solidly validates the accuracy of the present method.

**Figure 3:**
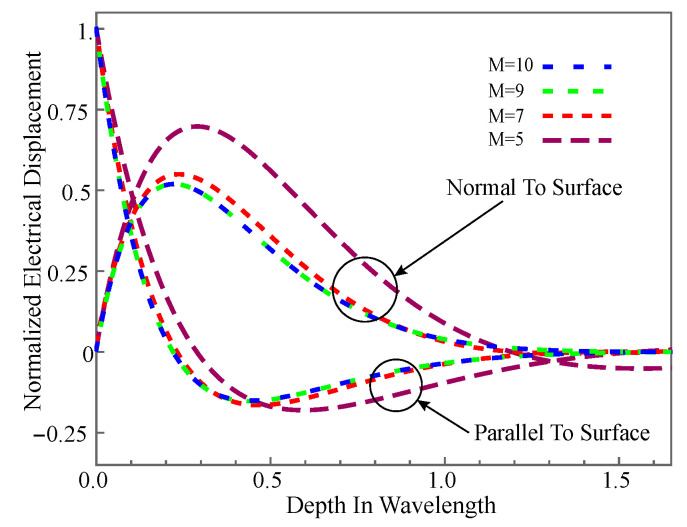
Normalized mechanical displacement amplitude distribution with different *M*.

Similarly, the normalized electrical displacement distributions are illustrated in Figure 3, in which its curves are normalized using the electric displacement on the upper surface. The figure gives the electric displacements of *M* at different values. As can be seen, although different *M* will generate different curves, their electric displacements all are zeros at z=0. This is consistent with the description of Equation (4). All these show that the boundary conditions are met well. On the other hand, the curves converge with *M* increases, and the result when *M* is 9 is exactly the same as when *M* is 10, which shows that the solutions have converged well.

### 3.2. Full Frequency Spectra

**Figure 4:**
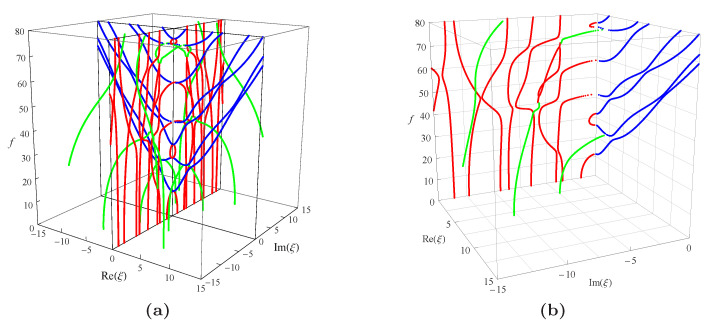
Full dispersion spectra of complex Rayleigh waves for a nonhomogeneous magneto-electro-elastic half-space: (**a**) 3D full frequency spectra; (**b**) one quarter of the 3D full frequency spectra. Nonhomogeneous coefficient n=1, $\xi =k\times 10^{2}$; blue branches for real solutions, red for imaginary, green for complex.

By examining Equation (19), one can see that, if a series of angular frequency values are given, a series of corresponding wave numbers will be generated in the form of eigenvalues. As is well known, ω/2π will always give the real frequency *f*. The wave numbers obtained in the present paper can be categorized into three kinds of different roots: the purely real, the pure imaginary, and the complex. Taking the real wavenumbers as the *x*-axis, imaginary wavenumbers as the *y*-axis, and *f* as the *z*-axis, the full frequency spectra for a nonhomogeneous magneto-electro-elastic half-space will be constructed just like Figure 4. $\xi =k\times 10^{2}$ in this figure is available everywhere in the present paper and will not be explained anymore.

Figure 4a presents the full frequency spectra with the nonhomogeneous coefficient n=1 in the function $f\left(z\right)={\left(1+z\right)}^{n}$, the material constants used to calculate in the figure is listed in Table 1. Which shows that the wavenumber roots occur in pairs of opposite signs, i.e., if a+bi(a,b∈R) is a solution of Equation (19), then ±a±bi all will be the solutions of it, and they are all distributed symmetrically in the top four quadrants as illustrated in the figure. For b/a=0, the branches represent real waves (with blue dotted curves); For a/b=0, the branches represent non-propagating waves (with red dotted curves in the figure), in which amplitudes decay exponentially; for ab≠0, the branches represent complex waves (with green dotted curves in the figure) traveling in the exponentially damped trigonometric wave forms with propagating distance. In 3D complex space, the purely real branches generally show the trajectories of first decline and then rise, while the purely imaginary and complex branches are very complicated. They do not show obvious regularity, and some of them are converted into propagating modes.

**Figure 5:**
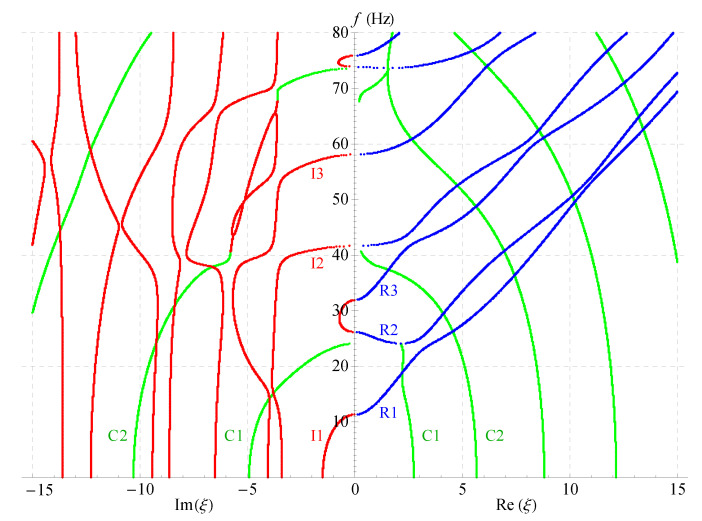
Two-dimensional full frequency spectra (n=1).

Considering the symmetry of the wave’s solution, here, we only present one quarter of its curves as shown in Figure 4b, then project all the curves onto the imaginary and real planes, respectively, as shown in Figure 5. It can be found that in this figure: For the purely imaginary branches, the first three branches I1, I2, and I3 are joined to purely real branches smoothly where the wavenumbers become zero; the purely imaginary branches become more complicated with the increase of frequency and the decrease of wavenumber. For the complex branches, some branches convert to purely real modes and continue propagating just as C1 branch; some branches convert to purely imaginary modes with the increase of frequency just as C2 branch. The real branches corresponding to propagating waves have been studied very deeply, so they do not necessitate the time to be discussed here. In a word, Figure 5 not only presents the values of wavenumbers and frequencies of the wave’s solution in the nonhomogeneous magneto-electro-elastic half-space, but it also clearly illustrates the relationship between the three kinds of solutions.

**Figure 6:**
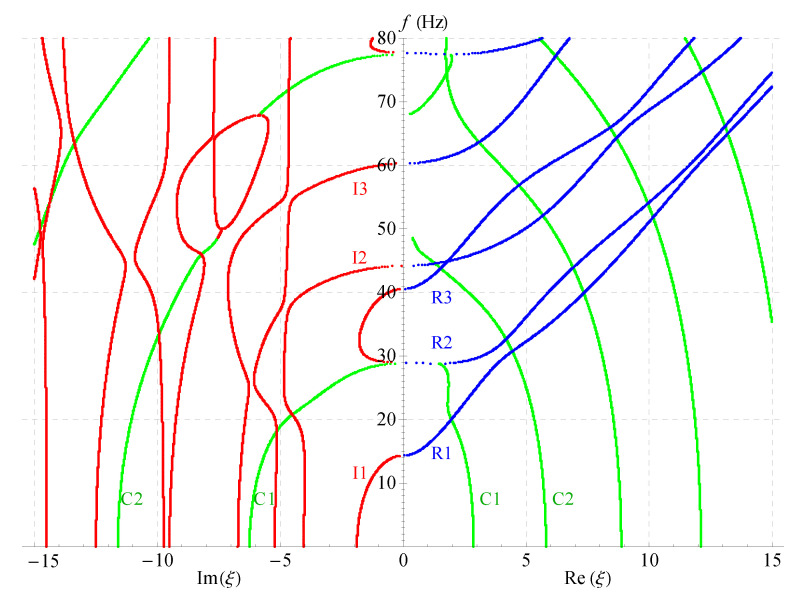
Two-dimensional full frequency spectra (n=2).

**Figure 7:**
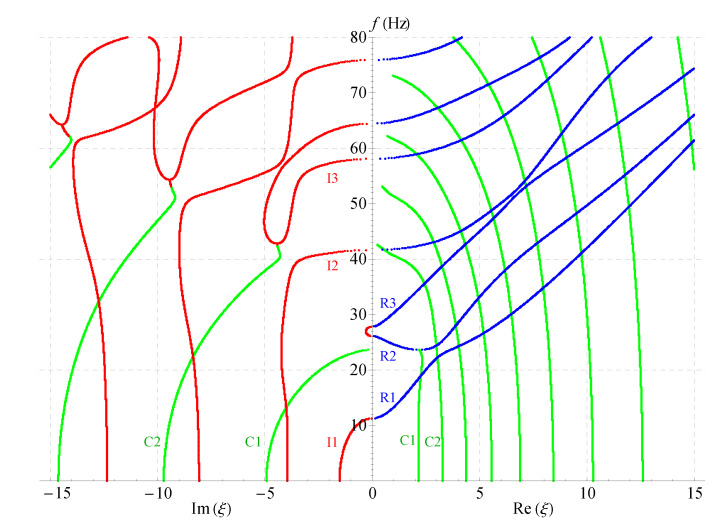
Two-dimensional full frequency spectra without considering magnetic field (n=1).

**Figure 8:**
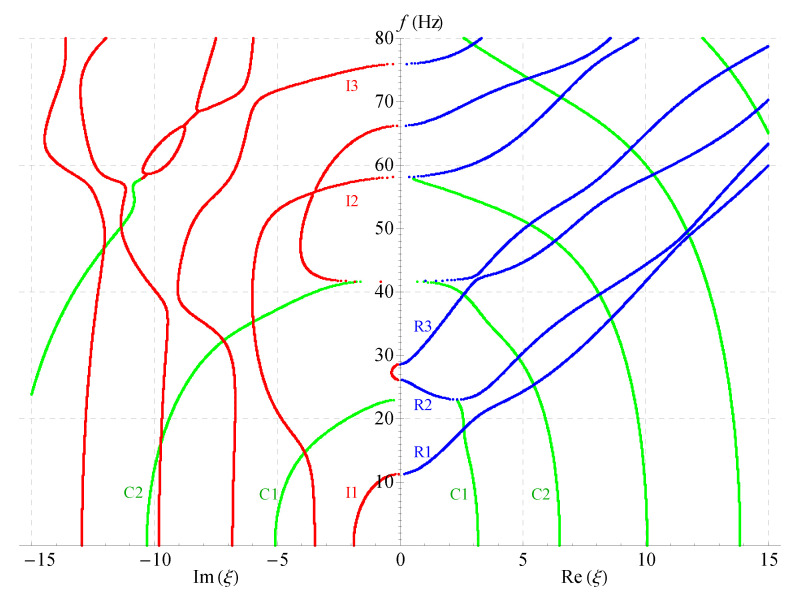
Two-dimensional full frequency spectra without considering electric field (n=1).

Figure 6, Figure 7 and Figure 8 present the 2D frequency spectra for the nonhomogeneous magneto-electro-elastic half-space with different cases. Their material constants all come from Table 1, but there exist differences in the nonhomogeneous, electric and magnetic coefficients. The material constants in Figure 6 are the same as that in Figure 5, except for the nonhomogeneous coefficient n=2; Figure 7 shows the frequency spectra without the magnetic field, and the material used in Figure 8 without the electric field. All waves propagate in the *x*-axis and their thickness directions are all along *z*-axis in these figures. The same as in the 3D figures, the point curves in blue correspond to real solutions, those in red correspond to imaginary solutions, and green to complex.

Comparing Figure 5, Figure 6, Figure 7 and Figure 8 demonstrates that: nonhomogeneous coefficients have great influence not only on the cut-off frequencies of propagating waves, but, also on those of the complex and imaginary waves, the cut-off frequencies increase as the nonhomogeneous coefficients increase, e.g., the cut-off frequency of R1 branch is 24 when nonhomogeneous coefficient is 1, but it becomes 29 when the nonhomogeneous coefficient increases to 2; the cut-off frequency difference between the two branches R2 and R3 becomes larger with the increasing of nonhomogeneous coefficients, so that R3 crosses with the fourth branch. However, the electric or magnetic coefficients have little effect on the cut-off frequencies, which is verified in Figure 7 and Figure 8. All these show that the influence of nonhomogeneous coefficients on cut-off frequencies is more significant than that of magnetic or electric coefficient.

Another finding is: there are more branches of green curves appeared in the right half field of Figure 7, while the quantity of this color curves has barely changed in the left half field compared with other three figures. This means that the ratio Im(ξ)/Re(ξ) of the complex solution becomes larger in this case. This indirectly shows that the magnetic coefficients have notable effect on the ratio. However, the ratio almost does not change in Figure 6 and Figure 8. Therefore, the ratio Im(ξ)/Re(ξ) of the complex wave number can be changed by changing the volume fraction of the magnetic part in a composite material. The numbers of the imaginary branches of the complex solution (green curves in the left half field of figure) are basically the same in Figure 5, Figure 6, Figure 7 and Figure 8, which shows that the magnetic, electric and nonhomogeneous coefficients have little effect on their imaginary part of the complex solutions in the magneto-electro-elastic nonhomogeneous half-space.

### 3.3. The Analysis of Displacement and Field Distributions

To explore the complex wave structures in the nonhomogeneous magneto-electro-elastic half-spaces, this section will present their displacement, magnetic, and electric potential field distributions, at the same time compare them with those of the propagating waves. Here, we examine the points in the close vicinity f=24Hz, where is the transition area from complex waves to propagating waves, which can be seen in Figure 4 and Figure 5. The exact solution can be obtained by the following steps: firstly, deriving the eigenvector R and wavenumber *k* from Equation (19), and immediately getting the numerical representation of vector P in Equation (14); and further getting the displacement, magnetic, and electric potential field distributions by Equations (9) and (11).

**Figure 9:**
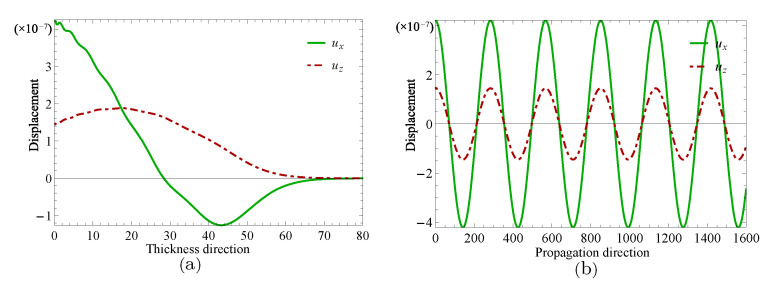
Displacement distributions for thickness direction in (**a**) and propagation direction in (**b**) when f=24.10Hz,ξ=2.2152.

**Figure 10:**
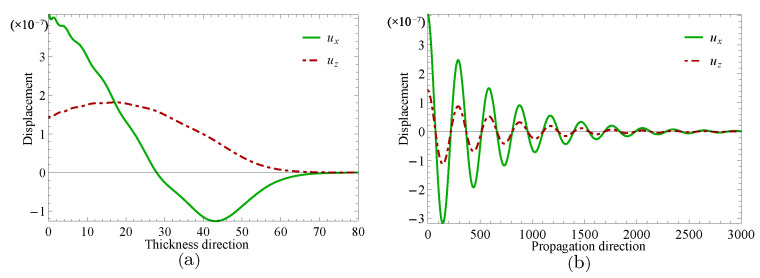
Displacement distributions for thickness direction in (**a**) and propagation direction in (**b**) when f=24.07Hz,ξ=2.1365+0.1694i.

**Figure 11:**
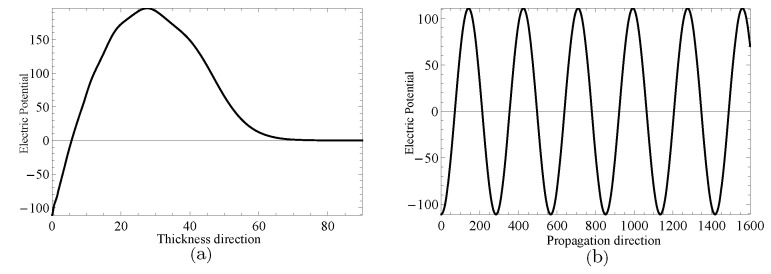
Electric potential distributions for thickness direction in (**a**) and propagation direction in (**b**) when f=24.10Hz,ξ=2.2152.

**Figure 12:**
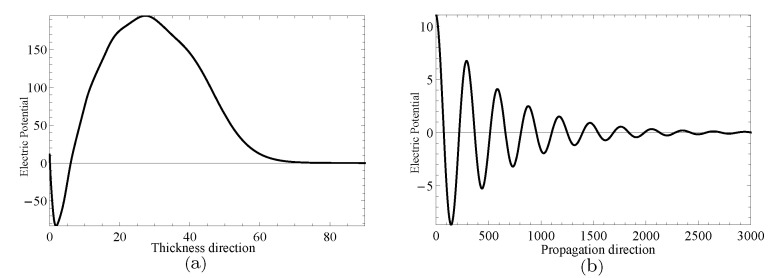
Electric potential distributions for thickness direction in (**a**) and propagation direction in (**b**) when f=24.07Hz,ξ=2.1365+0.1694i.

**Figure 13:**
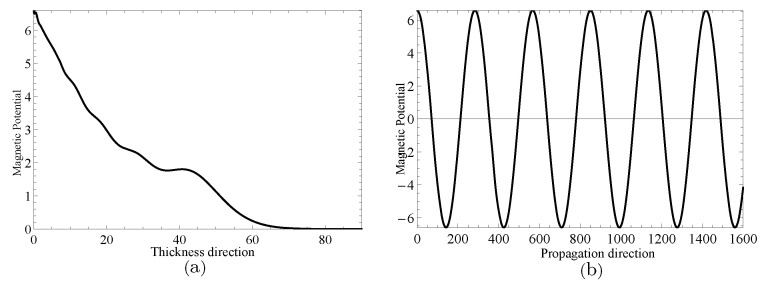
Magnetic potential distributions for thickness direction in (**a**) and propagation direction in (**b**) when f=24.10Hz,ξ=2.2152.

**Figure 14:**
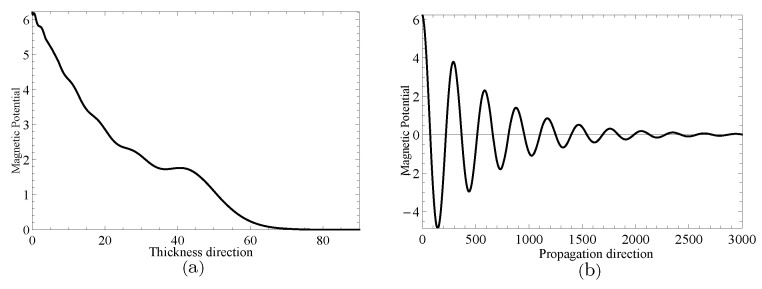
Magnetic potential distributions for thickness direction in (**a**) and propagation direction in (**b**) when f=24.07Hz,ξ=2.1365+0.1694i.

Next, two specific cases will be given to investigate the complex wave structures, their material coefficients are given in Table 1 and their nonhomogeneous coefficient n=1. One is ξ=2.2152, when f=24.10Hz, and another is ξ=2.1365+0.1694i, when f=24.07Hz, which can be obtained through the method mentioned in the previous paragraph. Figure 9 and Figure 10 show the displacement distributions along *z* and *x* in Figure 1, Their subfigure (**a**) are obtained at *x* is zero and (**b**) at *z* is zero, when f=24.10Hz,ξ=2.2152 and f=24.07Hz,ξ=2.1365+0.1694i, repectively. Figure 11 and Figure 12 present the electric potential distributions, Figure 13 and Figure 14 the magnetic potential distributions. The result, by comparing Figure 9a and Figure 10a, this illustrates that there is barely change of the mechanical displacement field distributions in the thickness direction when the complex wave approaches to the propagating wave, and the similar phenomenon is also found in electric and magnetic potential distributions in the thickness direction. Figure 9a, Figure 10a, Figure 11a, Figure 12a, Figure 13a and Figure 14a demonstrate that the displacement, electric, and magnetic potential all decay to their final zero points at about z=75, even though the attenuation trajectories of mechanical displacement are obviously different from that of the electric and magnetic potential. Figure 9b, Figure 10b, Figure 11b, Figure 12b, Figure 13b and Figure 14b show that: for complex waves, the electric and magnetic potential, same as in displacement, decay in the propagation direction *x* following a damped sinusoidal distribution rather than only exponential decay; the complex waves are able to travel farther and farther with their approaching purely real solution.

### 3.4. Effects of Nonhomogeneous and Magnetoelectric Properties on Phase Velocity

**Figure 15:**
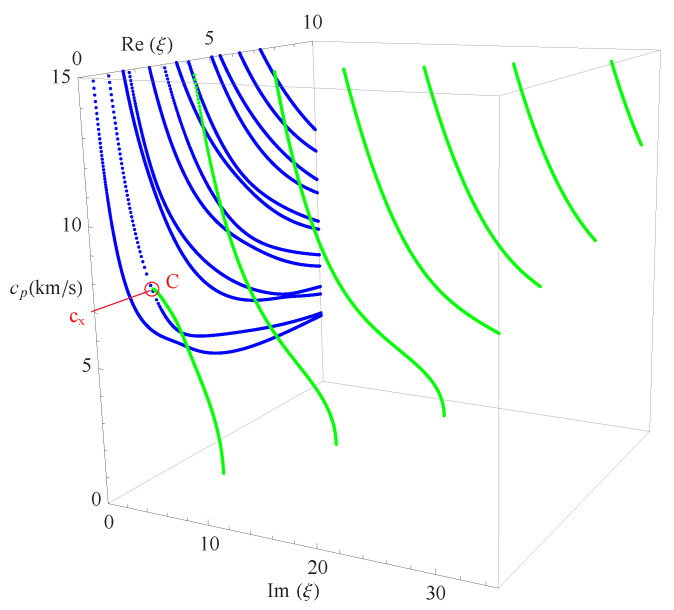
Three-dimensional phase velocity curves of the complex waves, blue branches for purely real solutions, and green for complex.

**Figure 16:**
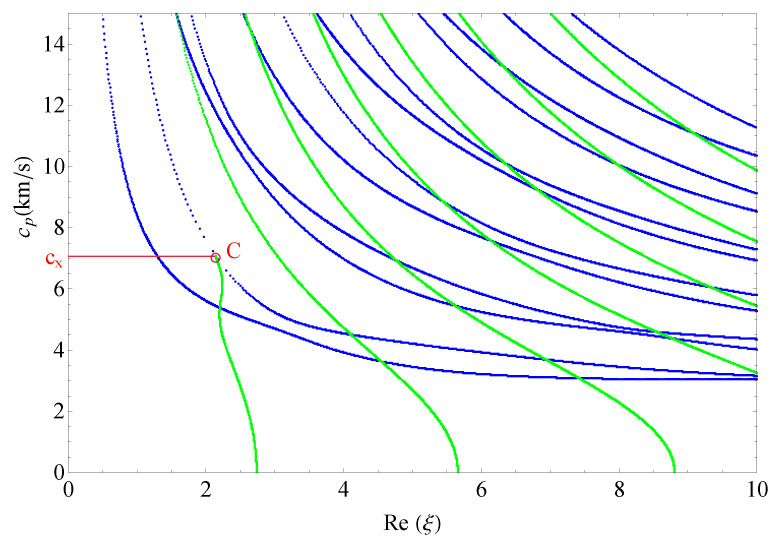
The projection of 3D phase velocity curves, blue branches for purely real solutions, and green for complex.

This section presents the phase velocities of complex waves for different cases, further discusses the effects of nonhomogeneous, magnetic, and electric coefficients on phase velocity. The phase velocity is defined as $c_{p}=\omega /Re\left(\xi \right)$ in the present paper. Figure 15 presents the 3D phase velocity curves for a nonhomogeneous magneto-electro-elastic half-space. In this figure, the real ξ is set to *x*-axis, the imaginary ξ is set to *y*-axis, and the phase velocity $c_{p}$ is set to *z*-axis; then, the purely real modes and the complex modes can be plotted in a three-dimensional coordinate system with ease, but the purely imaginary modes are not drawn here because their corresponding waves cannot propagate. The 3D phase velocity curves not only clearly present the purely and complex wave modes but also describe the process that the complex waves degenerate to purely real wave modes, e.g., the marked *C* point in Figure 15 illustrates where the complex wave mode converts to purely real mode. To express the phase velocity of converting point more clearly and facilitate the subsequent comparisons with different cases, here, we project all the curves in Figure 15 to $Re\left(\xi \right)-c_{p}$ plane, as shown in Figure 16. As a result, the phase velocity $C_{x}$ and the converting point *C* can be easily marked and are suitable to compare.

**Figure 17:**
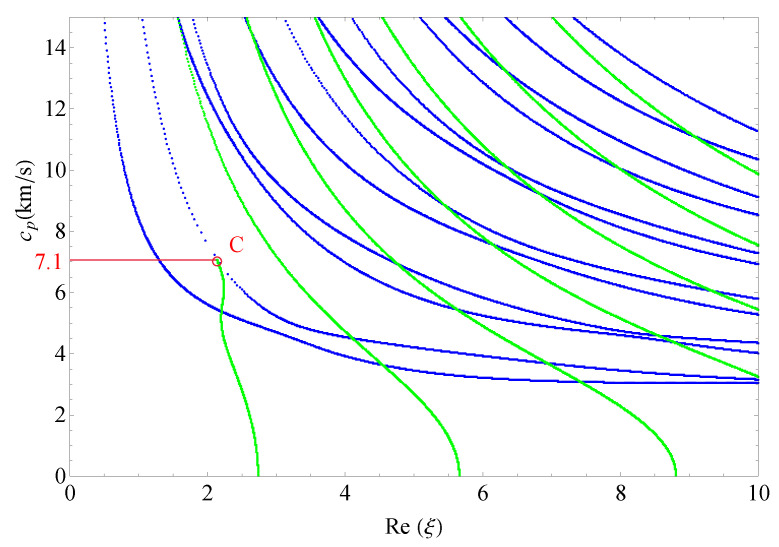
Phase velocity spectra (n=1).

**Figure 18:**
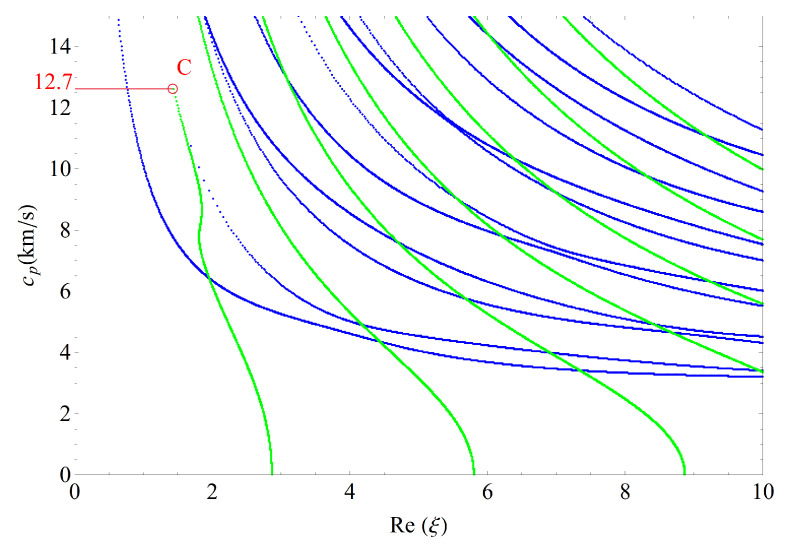
Phase velocity spectra (n=2).

**Figure 19:**
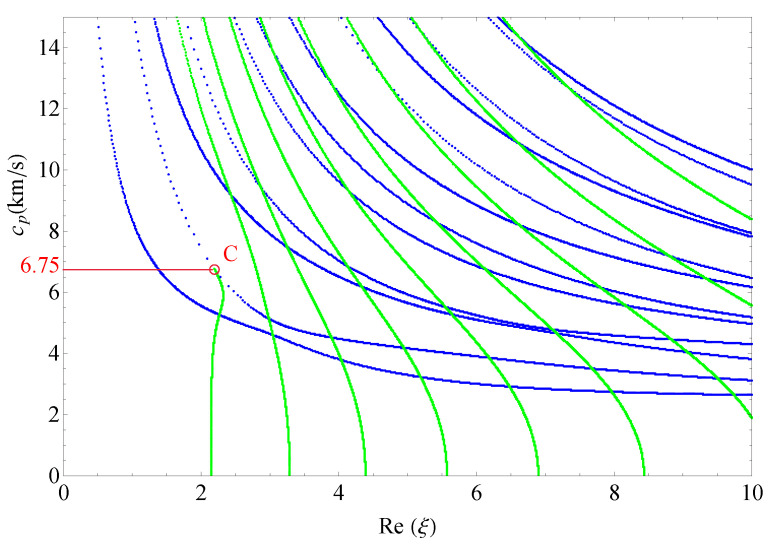
Phase velocity spectra without magnetic field (n=1).

**Figure 20:**
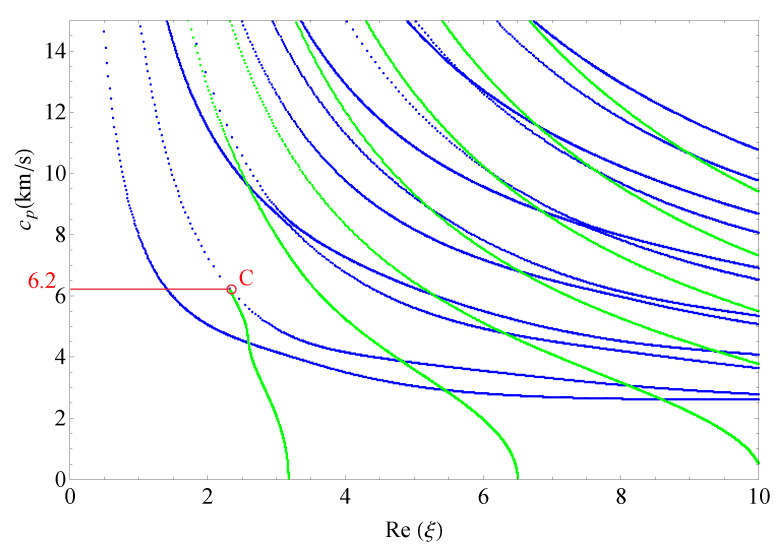
Phase velocity spectra without electric field (n=1).

Figure 17 shows the 2D phase velocity curves for the nonhomogeneous magneto-electro-elastic half-space, the result obtained at the nonhomogeneous coefficient n=1 with the applied material constants taken from Table 1, which illustrates that the phase velocity of the converting point is 7.1/s. Figure 18, Figure 19 and Figure 20 present the phase velocity curves with different cases. Figure 18 describes the phase velocity curves same as Figure 17, but, for nonhomogeneous coefficient n=2, Figure 19 gives the phase velocity curves same to as Figure 17, but excluding the magnetic constants, and Figure 20 excluding the electric constants. It can be seen that the phase velocity of converting point soars to 12.7/s when the nonhomogeneous coefficient n=2, which illustrates that nonhomogeneous coefficient has significant effect on converting point. Figure 19 presents the frequency spectra in the nonhomogeneous piezoelectric half-space, in which the phase velocity of converting point is 6.75km/s near to 7.1km/s, so magnetic coefficients have little effect on the converting point. The case in Figure 20 has similar characteristics to the previous case with respect to the converting point, but the value of converting point goes down slightly instead of increasing. Therefore, changing nonhomogeneous coefficient is the most straightforward method of changing the phase velocity of converting point, compared with changing electric or magnetic coefficients.

## 4. Conclusions

The Laguerre orthogonal polynomial expansion approach was improved to investigate the complex Rayleigh waves propagating in the nonhomogeneous magneto-electro-elastic half-spaces. The proposed method can present not only the solution of propagation waves but also that of complex or evanescent waves. The following conclusions can be drawn according to the numerical examples:

1. The developed method takes angular frequency as independent variable and constructs characteristic equation, furthermore transforms the problem of finding the solution of acoustic waves into an eigenvalue problem, and successfully obtains the real, imaginary and complex wave modes.
2. In this configuration and material, although electric and magnetic properties have less effect on the cut-off frequencies of complex solutions than nonhomogeneous coefficient, magnetic properties have more effect on the ratio of the real part to the imaginary part of complex solutions than other material properties.
3. The energies of mechanical, electric and magnetic fields will decay to zero after traveling to the same point in the thickness direction, but the attenuation trajectories of mechanical displacement are obviously different from those of electric and magnetic potential. In the propagation direction, displacement, electric, and magnetic potential have similar attenuation tendencies and converting process.
4. Nonhomogeneous coefficient has significant effect on the phase velocity of complex converting point, but the electric and magnetic properties do not, which, on the contrary, make the velocity slightly smaller.

The proposed method can be extended to other more complicated nonhomogeneous conditions with ease, even though here employed only one nonhomogeneous function for the convenience of expression. In practice, the function can be any derivative continuous function, so giving distinct nonhomogeneous function, respectively, to different material coefficient at the same time will make the present method more universe. Moreover, the propagation characteristics and behaviors of Rayleigh waves in layered half-space play an important role in nondestructive evaluation, ocean acoustics, seismic prospecting, etc., so further attention will be paid on the complex surface waves propagating in a layered half-space.

## Figures and Tables

**Table 1:** Material properties of the nonhomogeneous magneto-electro-elastic half-space [40] at z=0.

Item (Unit)	Symbol & Value				
Elastic constants (GPa)	$c_{11}$	$c_{33}$	$c_{44}$	$c_{12}$	$c_{13}$
	139	115	25.6	77.8	74.3
Piezoelectric constants ($C/m^{2}$)	$e_{31}$	$e_{33}$	$e_{15}$		
	−5.2	15.1	12.7		
Dielectric constants ($\times 10^{-9}\;F/m$)	$\epsilon_{11}$	$\epsilon_{33}$			
	6.46	5.62			
Piezomagnetic constants (N/Am)	$q_{31}$	$q_{33}$	$q_{15}$		
	580.3	699.7	550		
Magnetic constants ($\times 10^{-6}\;{\mathrm{Ns}}^{2}/C^{2}$)	$\mu_{11}$	$\mu_{33}$			
	5	10			
Electromagnetic constants ($\times 10^{-11}\;\mathrm{Ns}/\mathrm{VC}$)	$d_{11}$	$d_{33}$			
	−3612.68	−2.4735			

## Data Availability

The data presented in this study are available on request from the corresponding author.
